# Right-Sizing Testing Before Elective Surgery for Patients With Low Risk

**DOI:** 10.1001/jamanetworkopen.2025.35750

**Published:** 2025-10-06

**Authors:** Nicole M. Mott, Dana Greene, Erin Kim, Valerie Mefford, Anthony Cuttitta, Faelan Jacobson-Davies, Shawna N. Smith, Eve A. Kerr, Anthony L. Edelman, Michael Mathis, Michael Englesbe, Hari Nathan, Lesly A. Dossett

**Affiliations:** 1 National Clinician Scholars Program, University of Michigan, Ann Arbor; 2 Veterans Affairs Center for Clinical Management Research, Ann Arbor, Michigan; 3 Center for Healthcare Outcomes and Policy, University of Michigan, Ann Arbor; 4 Michigan Program for Value Enhancement, Ann Arbor; 5 University of Michigan Medical School, Ann Arbor; 6 University of Michigan School of Public Health, Ann Arbor; 7 Department of Internal Medicine, University of Michigan, Ann Arbor; 8 Department of Anesthesiology, University of Michigan, Ann Arbor; 9 Department of Surgery, University of Michigan, Ann Arbor

## Abstract

**Importance:**

Guidelines recommend against testing before low-risk surgery in healthy patients because it offers no benefit and may cause harm. However, testing remains prevalent, highlighting the need for a deimplementation strategy that can be broadly applied across health care settings.

**Objective:**

To assess the feasibility of a multifaceted, multicomponent deimplementation strategy entitled Right-Sizing Testing Before Elective Surgery (RITE-Size), hypothesizing it would be feasible to execute with 80% of milestones met on time.

**Design, Setting, and Participants:**

This quality improvement study was conducted from March 1 to August 31, 2024, at 3 hospitals of varying characteristics in Michigan. The intervention was structured into 3 phases (baseline, preparation, and active deimplementation) and further divided into 6 milestones (ie, key steps in the deimplementation process). Eligible preoperative tests included bloodwork and cardiopulmonary evaluations (eg, blood cell counts, metabolic panels, chest radiography, and electrocardiography) performed within 30 days of elective laparoscopic cholecystectomy, inguinal hernia repair, or breast lumpectomy in healthy adults.

**Interventions:**

The intervention included site visits, coaching sessions, data review, initiation of consensus processes for deimplementation, and distribution of strategy components (eg, decision support tools).

**Main Outcomes and Measures:**

The primary outcome was milestone completion. Secondary outcomes included acceptability and appropriateness, as assessed by the Acceptability of Intervention Measure (AIM) and the Intervention Appropriateness Measure (IAM). Additionally, barriers and facilitators to implementation were evaluated through semistructured interviews, along with testing rates derived from claims data.

**Results:**

A total of 203 patients (mean [SD] age, 57 [17] years; 117 [57.6%] female) who underwent procedures of interest were identified. All milestones were achieved on time. The intervention had high acceptability and appropriateness among stakeholders (median [IQR], 20 of 20 [18-20] for AIM and 20 of 20 [16-20] for IAM). Key facilitators included small, cohesive, perioperative teams and the incorporation of the intervention into policy, supported by auditing and feedback systems. Barriers included the need for ongoing education and coordination across large health care systems. Testing rates significantly decreased across all sites from 68.0% (51 of 75) to 40.3% (25 of 62) (*P* = .001).

**Conclusions and Relevance:**

This quality improvement study of a multifaceted, multicomponent deimplementation strategy to reduce unnecessary preoperative testing at diverse hospital sites demonstrated feasibility of expanding this work in a stepped-wedge cluster randomized trial. These results suggest that hospital systems can use this deimplementation strategy in the future to reduce unnecessary preoperative testing.

## Key Points

**Question:**

Can a multifaceted, multicomponent deimplementation strategy aimed at reducing unnecessary preoperative testing be implemented across health care settings?

**Findings:**

In this quality improvement study, a preoperative testing deimplementation strategy was successfully implemented at 3 hospital sites as demonstrated by timely milestone completion. Additionally, the intervention was deemed highly appropriate and acceptable by key stakeholders and resulted in a significant reduction in preoperative testing rates from 68.0% to 40.3%.

**Meaning:**

These findings suggest that this preoperative testing deimplementation strategy is feasible for implementation across various health care settings, offering a scalable strategy to decrease low-value preoperative testing before common, low-risk surgical procedures that is appropriate and acceptable among key stakeholders.

## Teaser

This quality improvement study assesses the feasibility and outcomes of a preoperative testing deimplementation strategy in 3 hospitals in Michigan.

## Introduction

Performing routine preoperative tests (eg, blood tests or electrocardiography) before low-risk surgical procedures in healthy patients does not change management or improve outcomes.^1^ However, it can lead to unintended harm.^2^ Unnecessary testing may initiate care cascades or a series of avoidable tests and procedures arising from incidental findings.^2,3,4^ Treatment delays may result as patients await additional preoperative workups before proceeding with low-risk operations.^2,5,6^ It also incurs excess costs, with estimates noting $18 billion spent annually on low-value preoperative testing and downstream care cascades.^2,7,8,9^ These costs burden not only the health care system but also patients, who may face copayments, coinsurance, and missed workdays.^2^

As a result, practice guidelines advise against routine preoperative testing for common, low-risk surgical procedures. Specifically, the Choosing Wisely campaign, in partnership with the Society of General Internal Medicine,^9,10^ the American Society of Anesthesiologists,^11^ and the United Kingdom’s National Institute for Health and Care Excellence,^12^ have all recommended against routine preoperative testing since the early 2010s. Nevertheless, preoperative testing remains common, with considerable variation at the hospital level.^13,14,15,16,17^ For example, in a statewide cohort of patients undergoing several low-risk procedures, more than half (52%) had at least 1 low-value preoperative test performed, with hospital testing rates varying widely from less than 20% to more than 80% of cases.^13^ Similarly, national data from the American College of Surgeons National Surgical Quality Improvement Program indicates that preoperative testing occurred for most patients (>50%), with little reduction in rates after the release of the aforementioned guidelines.^14,16^

Through preliminary work, we have investigated targets for deimplementing unnecessary preoperative testing and demonstrated success in reducing testing within a single hospital system.^18,19,20^ However, individual interventions have not been integrated into a comprehensive deimplementation strategy for diverse general surgery procedures or systematically used across various health care settings. Therefore, in this quality improvement study, we aimed to conduct a pilot feasibility study of a multifaceted deimplementation strategy entitled Right-Sizing Testing Before Elective Surgery (RITE-Size). The objective was to test and refine the deimplementation strategy across 3 hospitals in Michigan before a planned stepped-wedge cluster randomized trial.

## Methods

We conducted a quality improvement study at 3 hospital sites in Michigan from March 1 to August 31, 2024. The primary outcome was the timely completion of 6 study milestones across 3 phases. We hypothesized that the intervention would be feasible to execute, with more than 80% of milestones being met on time. The study design and results are reported in accordance with the Standards for Quality Improvement Reporting Excellence (SQUIRE) reporting guideline^21^ and incorporate best-practice principles for implementing pilot studies.^22^ The University of Michigan institutional review board determined that this study aligns with quality improvement efforts and is therefore not regulated or subject to ongoing review or informed consent requirements.

### Hospital Sites and Statewide Quality and Value Partnerships

The hospital sites selected included institutions of various types, sizes, patient demographics, and geographic locations. Eligible sites were required to have preoperative testing rates of at least 50% for the procedures under consideration. Site 1 was a community hospital located in a city with approximately 11 000 people. Site 2 was a community hospital in a city with a population of approximately 20 000 people. Site 3 was a regional academic affiliate hospital in a city with approximately 110 000 people.

The sites participating in RITE-Size were simultaneously involved in 1 of 2 statewide quality and value partnership programs: the Michigan Value Collaborative (MVC) and the Michigan Surgical Quality Collaborative (MSQC). MVC is a Blue Cross Blue Shield of Michigan (BCBSM)–sponsored value collaborative that includes 105 hospitals and 33 physician organizations throughout Michigan. MVC maintains a validated, prospectively collected claims registry, encompassing BCBSM Commercial Preferred Provider Organization (PPO), BCBSM PPO Medicare Advantage, Blue Care Network (BCN), BCN Medicare Advantage, Medicare Fee for Service, and Michigan Medicaid. MSQC is a BCBSM-sponsored surgical quality collaborative consisting of more than 70 hospitals with overlapping membership with MVC. MSQC maintains a prospectively collected database containing facility- and surgeon-specific data on key quality indicators of interest following a representative sampling algorithm and managed by trained data abstractors.

During the study period, MVC and MSQC offered distinct voluntary quality improvement initiatives that included a pay-for-performance scorecard awarding points for participating in efforts to reduce preoperative testing and for reducing testing rates.^23,24,25,26^ Site 1 participated in the MVC initiative, and sites 2 and 3 participated in the MSQC initiative. The aim of the current study is to analyze the feasibility of the RITE-Size intervention and not the pay-for-performance incentive structure.

### Preoperative Tests, Patients, and Surgical Procedures

Eligible preoperative tests performed within 30 days of surgery included the following: electrocardiography,^11,27^ transthoracic echocardiography, cardiac stress test, chest radiography, urinalysis, complete blood cell count, basic metabolic panel, comprehensive metabolic panel, coagulation studies, and pulmonary function tests, including blood gas analysis and spirometry. Data were collected from patients 18 years or older. Demographic variables, including race and ethnicity, were identified through claims data and were collected for readers to determine generalizability to their own practice settings. Categories are denoted exactly as they appear in the database for the single race and ethnicity variable: Asian or Pacific Islander, Black, Hispanic, White, and other or unknown. The races and ethnicities included in the other group are not specified in the data. Patients needed to be classified as American Society of Anesthesiology class I or II, thereby excluding patients with major medical comorbidities that would increase surgical risk. Patients were excluded if they had visited an emergency department within the 30 days before surgery to exclude instances of potentially clinically appropriate testing for evaluation of acute symptoms.

Eligible surgical procedures included elective outpatient laparoscopic cholecystectomy, inguinal hernia repair, or breast lumpectomy, as defined by *Current Procedural Terminology* codes. These procedures were chosen because they are common, low-risk elective procedures likely to be performed by a single group of clinicians (ie, general surgeons), but they vary in patient mix based on key attributes associated with variation in preoperative testing, such as age and comorbidities. However, routine preoperative testing without specific clinical indications is not recommended for any of these procedures.^9,10,11^

### Deimplementation Strategy Components

We developed RITE-Size, a multilevel, multicomponent deimplementation strategy based on the process of deimplementation proposed by Niven et al.^28^ Theory-informed deimplementation strategies were selected through a literature review and preliminary investigations, incorporating qualitative work and focused ethnography that explored key barriers and facilitators to the deimplementation of low-value preoperative testing from the perspectives of various stakeholders.^18,29^ Determinants were mapped to general deimplementation strategies using the Tailored Implementation for Chronic Disease (TICD) checklist, which consolidates multiple domains, including guidelines, health professionals, patients, organizations, policy, and legal factors (Table 1).^30,31,32^

**Table 1.** TICD Determinants Paired With Deimplementation Strategies to Reduce Low-Value Preoperative Testing Through a Multifaceted, Multicomponent Intervention

TICD determinant	TICD definition	Application to low-value, preoperative testing	Strategy
Expected outcome	The extent to which professionals believe adherence will lead to the desired outcome	Clinicians overestimate the degree to which preoperative testing prevents adverse events	Provide education for clinicians on the downstream costs, harms, and impact on treatment decisions
Nature of the behavior	The degree of habit or automaticity of the behavior	Clinicians routinely order preoperative tests as part of outdated protocols or order sets	Provide decision support tools reminding clinicians of preoperative testing recommendations
Strength of supporters and opponents	The extent of support and opposition to necessary change	High-volume or influential clinicians may strongly agree or disagree with the recommendations	Lead a local consensus process to review and update low-risk preoperative testing pathways
Self-monitoring or feedback	The extent to which the professionals have the capacity for self-monitoring	Professionals may relapse to previous behaviors or forget to adhere	Provide clinician and facility-specific feedback of performance over time and benchmarked to statewide performance
Financial incentives	The extent to which patients, clinicians, and organizations have financial incentives to adhere	Facilities are not motivated to reduce overtesting because it leads to higher payments	Implement a pay-for-performance payment incentive rewarding facilities with low testing rates or prespecified improvement

Abbreviation: TICD, Tailored Implementation for Chronic Disease.

After identifying general deimplementation strategies, we tailored and implemented these strategies through an iterative process with clinician stakeholders, developing specific deimplementation components targeting multiple levels, including (1) in-person site visits, (2) implementation coaching sessions, (3) review of preoperative testing data, (4) consensus process with stakeholders to agree on the deimplementation strategy, and (5) distribution of strategy components (eg, modification of anesthesia protocols, use of decision support tools, and application of auditing and feedback). Components were designed to be implemented and adapted at local trial sites. Complementary to these components were the development of a website on appropriate preoperative testing,^33^ a decision aid (eAppendix 1 in Supplement 1), and a primary care engagement packet (eAppendix 2 in Supplement 1).

To determine feasibility, the research team developed 3 implementation phases divided into 6 milestones. In the baseline phase, members of the study team conducted in-person site visits (milestone 1) and followed up with an initial coaching session (milestone 2). The site visit included all key stakeholders, such as the head anesthesiologist and surgeon, the nurses who work in the preoperative testing space, and the quality lead alongside the RITE-Size team. The site visit covered the data supporting reduction of unnecessary preoperative testing and current guidelines regarding its use. It also was an opportunity for introductions, for the RITE-Size team to gain insight into the local site’s preoperative testing culture, and for highlighting the partnerships and resources available. The coaching session included the quality lead and the RITE-Size team and reviewed key information from the site visit with space for additional discussion. In the preparation phase, the study team held 2 additional implementation coaching sessions, which included a review of baseline preoperative testing data and a discussion of anticipated barriers or facilitators to prepare for active deimplementation (milestone 3). Based on these meetings, the sites created a protocol for the deimplementation of preoperative testing tailored to their local settings (milestone 4) and distributed it to eligible clinicians at each site (milestone 5). Milestones 4 and 5 were further supported by engagement with quality collaboratives (ie, MVC and MSQC). In the active deimplementation phase, the sites identified suitable clinicians for data collection to gather feedback on the deimplementation process (milestone 6). The research team remained available for ongoing support to help sites achieve their goals.

### Statistical Analysis

#### Data Collection and Quantitative Analysis

MVC claims data on hospital characteristics, patient characteristics, and preoperative testing rates were used. For this analysis, MVC provided insurance claims data from the following commercial payer sources: BCN, BCBSM Commercial PPO, BCN Medicare Advantage, and BCBSM PPO Medicare Advantage. We used descriptive statistics to examine on-time milestone completion. We calculated testing rates and nonparametric tests for trends to analyze binary testing rates. Where applicable, small count sizes in the tables were censored to comply with MVC data use guidelines. Data analysis was supported by Stata, version 18 (StataCorp). A 2-sided *P* < .05 was used to determine statistical significance.

#### Semistructured Interviews and Qualitative Analysis

In line with milestone 6, semistructured interviews (eAppendix 3 in Supplement 1) were conducted with key stakeholders at the 3 sites following the TICD framework to assess barriers and facilitators to deimplementing low-value preoperative testing as part of the RITE-Size intervention. Stakeholders were identified from individuals present at the site visit and key representatives from anesthesiology, surgery, nursing, and quality improvement departments. The interviews were transcribed verbatim, deidentified, and independently coded by 2 researchers (N.M.M., E.K.) using an inductive-deductive approach, with discrepancies resolved through consensus. A content analysis was conducted to map the determinants of testing and components of RITE-Size implementation to the TICD checklist. The interviews were supplemented by a survey (eAppendix 4 in Supplement 1) containing validated implementation outcome measures to evaluate acceptability and appropriateness, namely, the Acceptability of Intervention Measure (AIM) and the Intervention Appropriateness Measure (IAM).^34^

## Results

We identified 203 patients who underwent the procedures of interest across the sites (19 [9.4%] at site 1, 20 [9.9%] at site 2, and 164 [80.8%] at site 3), including 75 laparoscopic cholecystectomies, 77 inguinal hernia repairs, and 51 breast lumpectomies (Table 2). The mean (SD) age of the patient population was 57 (17) years, with 117 (57.6%) of the patients being female and 86 (42.4%) male (Table 2). Of the patients, 16 (7.9%) were Black, 158 (77.8%) were White, and 19 (9.4%) were of other or unknown race. All study milestones were completed on time at each site, achieving the stated hypothesis that more than 80% of milestones were met.

**Table 2.** Study Patient Characteristics (March to August 2024)^a^

Patient characteristic	No. (%) of patients^b^ (N = 203)
Age, mean (SD), y	57 (17)
Sex	
Male	86 (42.4)
Female	117 (57.6)
Race and ethnicity	
Asian or Pacific Islander	<10
Black	16 (7.9)
Hispanic	<10
White	158 (77.8)
Other or unknown^c^	19 (9.4)

^a^ Michigan Value Collaborative patient demographics for patients undergoing laparoscopic cholecystectomy, inguinal hernia repair, or breast lumpectomy at 3 pilot sites during the study period (March to August 2024).

^b^ Unless otherwise indicated. Cells with counts less than 10 were censored to comply with data use guidelines.

^c^ The races and ethnicities included in the other group are not specified in the data.

Nine stakeholders participated in semistructured interviews, including 3 hospital quality improvement leads, 2 preoperative testing nurses, 1 anesthesiologist, and 3 surgeons. Barriers included a lack of clarity surrounding the guidelines, the need for ongoing education to counter automated and ingrained testing behaviors, and challenges in coordinating across large health care systems or within institutional constraints. Facilitators included team cohesiveness and communication, strong leadership, incorporation of the intervention into anesthesia policy, and self-monitoring of progress through auditing and feedback systems. Regarding the latter, benchmarking a site’s preoperative testing rates against other sites in the surgical quality collaboratives was particularly motivating for change. The electronic medical record both aided and hindered implementation. Although it enabled sites to audit preoperative testing rates and provide feedback to clinicians, there were often setbacks in having to circumvent outdated or clinically inappropriate automated reminders to order tests. Representative quotations are mapped to the TICD framework in Table 3. RITE-Size received high acceptability and appropriateness scores among stakeholders, with a median (IQR) score of 20 of 20 (18-20) for AIM and 20 of 20 (16-20) for IAM.

**Table 3.** Representative Quotations Mapped to the TICD Framework

TICD framework component	Representative quotation
Guideline factors	“I think what was most helpful to us was having the weight of a collaborative behind a project.” (Participant 5)
	“We had to change anesthesia testing policy, but that’s a huge process—a yearlong evolution, really. Even though it’s a policy, it is in place for several months and individuals still [order tests] because it takes a while for that stuff to get cultured.” (Participant 8)
Individual health professional factors	“I didn’t feel like I had a whole lot of problems adapting to it myself. The other two nurses are still struggling with it a little bit—just with the mindset of we’re used to ordering so many tests. It’s hard not to at this point.” (Participant 1)
	“I think after changing the algorithm, the main hurdle that we had to go through is implementing it with the actual presurgical testing nurses because they’re just so used to doing things in one particular way. I guess that it’s just a habit. In a way, they’re not even really looking at the algorithms.” (Participant 9)
Professional interactions	“I think being that we’re such a small facility and a small group, it’s been an easier transition for us, maybe, than some of the bigger facilities. We are able to get together, brainstorm, work through things probably more than others.” (Participant 1)
	“I know we weren’t in every meeting, but I thought it was well done that we were involved in at least the main meetings…We didn’t really know we were over testing. So, bringing us into the process and gently handling us rather than telling us we were doing it wrong was good. I never felt offended.” (Participant 2)
Incentives and resources	“What really changed our practice was on a quarterly basis we get this MSQC [data report] where they compare your facility with [other] facilities. We realized that we were ordering too many tests compared to [other] facilities, so we used that argument to reduce testing.” (Participant 4)
	“No one wants to be the worst. So that’s always motivating for doctors, for sure. All I had to do was show them that [testing data], and they were on me daily about what we can do to fix it.” (Participant 5)
Capacity for organizational change	“It’s a bright time for us now because our anesthesiologist is a real go getter too. She’s more than willing to work with us on this.” (Participant 2)
	“I do think resources like having enough people to dedicate time to go into the charts, look at what’s being done, and then give that information back to the providers is difficult. We’ve had some eliminations in our hospital over the last year. So, people are tasked with more work and less time to do it.” (Participant 6)

Abbreviations: MSQC, Michigan Surgical Quality Collaborative; TICD, Tailored Implementation for Chronic Disease.

Mean testing rates across all sites significantly decreased during the study period from 68.0% (51 of 75) in months 1 to 2 to 37.9% (25 of 66) in months 3 to 4, to 40.3% (25 of 62) in months 5 to 6 (*P* = .001) Figure). There was site-level variation in the reduction of testing rates (Figure), aligning with the noted site-specific facilitators and barriers. For instance, site 1 had a small, cohesive perioperative team and a dedicated quality improvement lead; consequently, the site was able to implement changes rapidly, achieving the greatest absolute difference in testing rates. In contrast, site 3, being the largest hospital site, exhibited higher testing rates than the other sites for most of the study period, despite ultimately achieving a successful reduction in testing rates by the end of the study.

**Figure.**
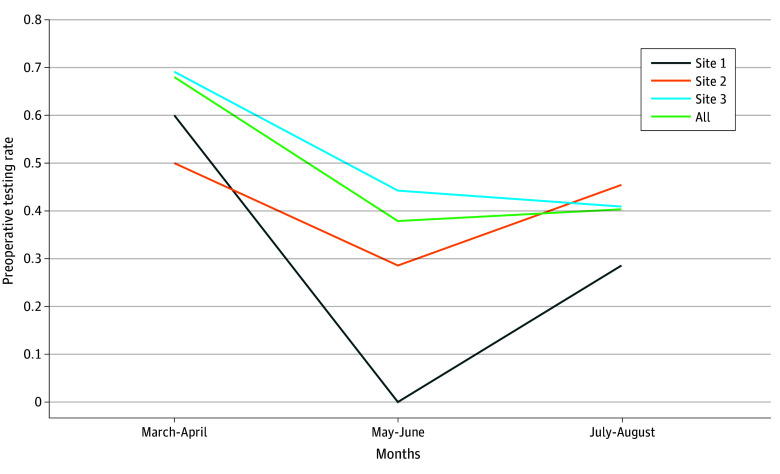
Michigan Value Collaborative (MVC) Testing Rates by Month Across All 3 Sites From March 2024 (Month 1) to August 2024 (Month 6)

## Discussion

We demonstrated success in this quality improvement study by on-time study milestone completion. We also showed high acceptability and appropriateness of the RITE-Size intervention among multidisciplinary stakeholders, alongside a reduction in unnecessary preoperative testing. Success was observed at all 3 pilot sites, which varied in institutional types, sizes, patient mixes, and geographic locations, thereby building on preliminary work that indicated the intervention’s favorable outcomes at a single academic institution.^20^ Collectively, these findings establish the foundation for a planned stepped-wedge cluster randomized trial across the state, ultimately aiming to minimize low-value preoperative testing before common, low-risk surgical procedures.

Although the multifaceted, multicomponent intervention was feasible, the insights from this study will be used to enhance the RITE-Size intervention. For instance, engaging stakeholders is a key component of successful evidence-based guideline implementation^35^; however, heterogeneity in approaches is reported in the literature.^35^ The RITE-Size team engaged with key stakeholders during the site visit and again when collecting feedback at the end of the intervention. Between these touchpoints, the RITE-Size team mainly collaborated with the quality lead at each site, who in turn communicated important information and implemented strategies with their team members. When gathering feedback at the conclusion, it became clear that some individual clinicians (eg, nurses, surgeons, or anesthesiologists) for whom the intervention was intended were unaware of all the RITE-Size efforts, resources, or strategies. Moreover, the relative influence of supporters and opponents of the intervention impacted its success. To better prepare clinicians, understand buy-in, and customize implementation strategies, in the next phase we will have relevant clinicians at each site complete the Organizational Readiness to Change Assessment^36^ instrument and use the results to inform implementation coaching sessions. Increasing engagement across all staff in this manner may help to avoid bottlenecks in workflows and mitigate reliance on key staff, as supported by previous implementation work for a large, multisite hospital intervention.^37^ Additionally, we have created an implementation guide incorporating lessons from the pilot work, such as anticipated barriers,^35^ to assist sites in successfully developing and implementing their protocols.

The specific barriers and facilitators to the implementation of the RITE-Size intervention will also be used as RITE-Size expands to additional hospital settings. For instance, smaller hospital systems adapted more quickly to the intervention due to strong cohesion among a limited number of multidisciplinary practitioners involved in the perioperative space. Additionally, smaller hospital systems did not face lengthy administrative processes for implementing changes, such as updating decision aids or anesthesia protocols. In contrast, the larger hospital took more time to adapt to the intervention due to challenges in coordinating with numerous clinicians across various perioperative workflows and settings. As RITE-Size expands, it is possible that the intervention will provide a strong framework for sites, but it may need to be tailored to meet local needs. For example, larger hospital sites may require buy-in from more individuals than the stakeholders identified in this work and a longer timeline, particularly in the baseline and preparation phases. The application of the Framework for Reporting Adaptations and Modifications to Evidence-Based Implementation Strategies^38^ in future work will inform how the RITE-Size intervention was adapted at pilot sites to anticipate site-specific modifications as the work is expanded.

### Limitations

There are important limitations to consider when interpreting the findings of this study. The patient population included was mostly non-Hispanic White patients, consistent with the demographics in Michigan; however, other settings with more diverse patient populations may experience unique considerations to implementing RITE-Size that we could not explore in the current study. We demonstrated high acceptability and appropriateness of the intervention among a convenience sample of key stakeholders, but future work should explore attitudes toward the intervention on a broader scale, including all clinicians—and patients—who may be impacted by the intervention.

The RITE-Size intervention is supported by ongoing quality improvement efforts through MVC and MSQC. Of note, participation in pay-for-performance programs alone may not result in reductions in unnecessary testing; previous work has shown variation in sites’ performances, with some high-performing hospitals reducing testing rates markedly and other low-performing hospitals failing to reduce testing rates.^39^ Nevertheless, cross-site collaboration (such as that achieved through statewide surgical collaboratives) has been shown to improve implementation of large, multisite hospital interventions^37^ and was a factor in the current study. Although other states may lack surgical collaboratives, MVC and MSQC hospitals include diverse practices, ranging from urban to rural, academic to community, and high to low volume, that are likely to mirror other hospital systems. Additionally, the claims data used in the analysis are limited to commercial payers and the inherent limitations of claims data, such as the lack of granular clinical detail. It is possible that misclassification errors could have occurred in that some of the testing performed was for purposes other than preoperative evaluation; however, we would expect this misclassification error to be similar across sites and not to contribute significantly to the overall volume of preoperative testing at sites with rates greater than 50%. Additionally, the testing rates reported by MVC mirrored sites’ internal MSQC reports containing quality improvement registry data obtained through trained abstractors. Previous work in the preoperative testing space has also shown that these claims-based data mirror that obtained through systematic medical record review.^20^

## Conclusions

In this quality improvement study, a multifaceted deimplementation strategy to reduce unnecessary preoperative testing before common, low-risk surgical procedures was feasible to implement, providing support for expanding this work in a stepped-wedge cluster randomized trial across the state. Hospital systems can use this deimplementation strategy in the future to reduce unnecessary preoperative testing.

## Notes

Supplement 1. **Appendix 1.** Decision Aid **Appendix 2.** Primary Care Physician (PCP) Letter **Appendix 3. **Interview Guide **Appendix 4.** Survey

Supplement 2.
Data Sharing Statement
