# Supplementary material for: Right-Sizing Testing Before Elective Surgery for Patients With Low Risk
Source: JAMA Netw Open. 2025 Oct 6;8(10):e2535750. doi: 10.1001/jamanetworkopen.2025.35750 (PMC12501813; doi:10.1001/jamanetworkopen.2025.35750)
Supplement: Supplement 1. — Appendix 1. Decision Aid Appendix 2. Primary Care Physician (PCP) Letter Appendix 3. Interview Guide Appendix 4. Survey [file jamanetwopen-e2535750-s001.pdf]

Supplemental Online Content

Mott NM, Greene D, Kim E, et al. Right-sizing testing before elective surgery for patients with low risk. *JAMA Netw Open*. 2025;8(10):e2535750. doi:10.1001/jamanetworkopen.2025.35750

Appendix 1. Decision Aid
Appendix 2. Primary Care Physician (PCP) Letter
Appendix 3. Interview Guide
Appendix 4. Survey

This supplemental material has been provided by the authors to give readers additional information about their work.

© 2025 Mott NM et al. *JAMA Network Open*.

Appendix 1:

Decision Aid

© 2025 Mott NM et al. *JAMA Network Open*.

MPOG MULTICENTER PERIOPERATIVE OUTCOMES GROUP
MPrOVE MICHIGAN PROGRAM ON VALUE ENHANCEMENT
MSQC MICHIGAN SURGICAL QUALITY COLLABORATIVE
MVC Michigan Value Collaborative
This resource was developed in partnership with MPOG, MPrOVE, MSQC, and MVC.

Suggested Preoperative Testing Decision Aid for Low-Risk Surgeries

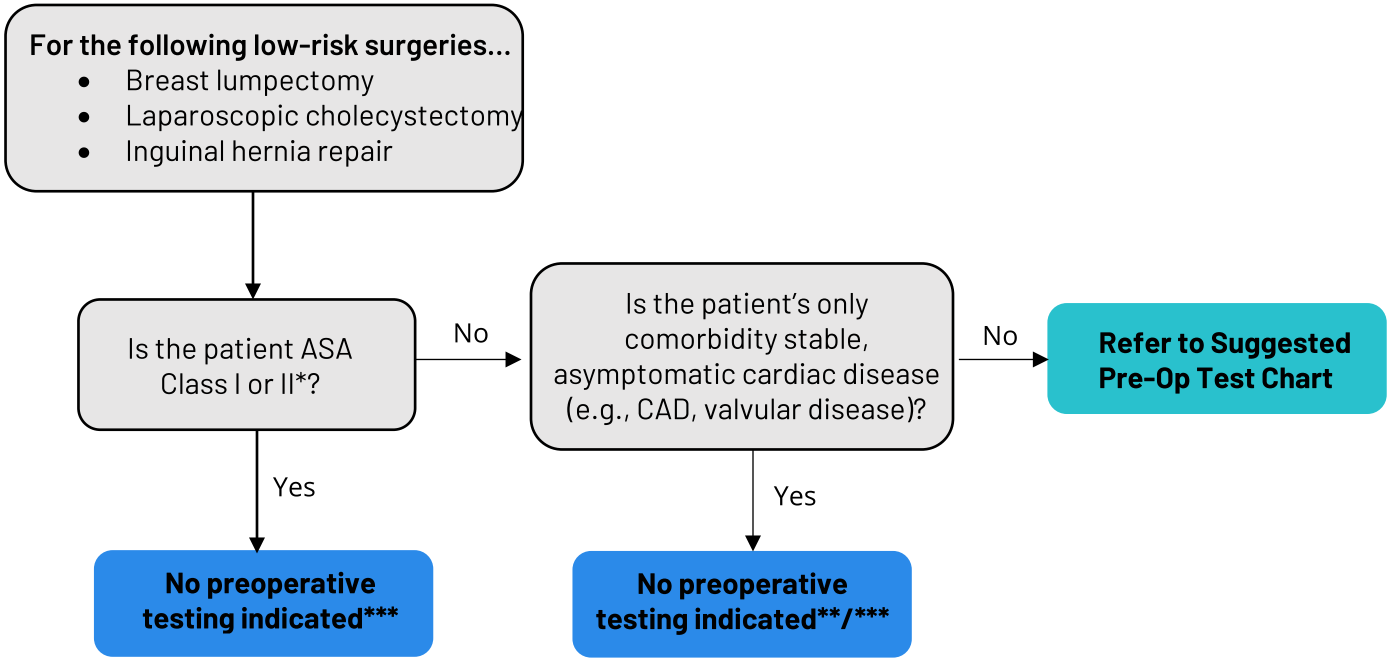

***American Society of Anesthesiologist (ASA) Physical Status Classification System:**

ASA Class I: Normal healthy patient. Non-smoking, no or minimal alcohol use, no acute or chronic disease, normal BMI.

ASA Class II: Mild systemic disease without substantive functional limitations. Current smoker, obesity (30<BMI<40), well-controlled DM/HTN, mild lung disease.

ASA Class III: Severe Systemic disease with substantive functional limitations, poorly controlled DM/HTN, COPD, morbid obesity (BMI ≥ 40), active hepatitis, alcohol dependence or abuse, pacemaker, moderate reduced EF, ESRD on dialysis, prior MI, CVA, TIA, or CAD/stents > 3 months ago

**May consider EKG if none available within the past ~6 months
***Except recent (3-6 months) potassium if on diuretic

All recommendations in this document pertain to non-pregnant, adult patients undergoing low-risk procedures. They do not replace clinical judgment and are intended as guidance only.

Version 1.4 February 2024

© 2025 Mott NM et al. *JAMA Network Open*.

Insert your hospital logo here

MPOG MULTICENTER PERIOPERATIVE OUTCOMES GROUP | MPrOVE MICHIGAN PROGRAM ON VALUE ENHANCEMENT | MSQC MICHIGAN SURGICAL QUALITY COLLABORATIVE | MVC Michigan Value Collaborative

This resource was developed in partnership with MPOG, MPrOVE, MSQC, and MVC.

Suggested Preoperative Tests for Patients Undergoing Low-Risk Surgery Who Are ASA III or Above

This chart does not replace clinical judgment and is intended as guidance only

	CBC	T&S	BMP	LFTs	INR/PT/PTT	EKG
**History of anemia, thrombocytopenia**	■					
**Cardiovascular disease**	■					■
**Anticoagulant use or history of bleeding disorder**	■	■			■	
**DM/major endocrine disease, prior electrolyte abnormalities, use of diuretics, antiarrhythmics, ACE/ARB**			■			
**Kidney disease**	■		■			
**Liver disease or risk of malnutrition**			■	■	■	
**Peripheral/cerebral vascular disease, cardiac risk factors, new cardiac symptoms**			■			■

CBC: Complete blood count
T&S: Type and screen
BMP: Basic metabolic panel
LFTs: Liver function tests
INR: International normalized ratio
PT: Prothrombin time
PTT: Partial thromboplastic time
EKG: Electrocardiogram
CXR: Chest radiography
ACE: Angiotensin-converting enzyme inhibitors
ARB: Angiotensin receptor blocker

References:

- Mocon A, McRitchie D, Tharani A. *Drop the Pre-Op: A toolkit for reducing unnecessary visits and investigations in pre-operative clinics.* Ontario, CA. 2019.
- Chow, W. B., Rosenthal, R. A., Merkow, R. P., Ko, C. Y., & Esnaola, N. F. (2012). Optimal preoperative assessment of the geriatric surgical patient: a best practices guideline from the American College of Surgeons National Surgical Quality Improvement Program and the American Geriatrics Society. *Journal of the American College of Surgeons, 215*(4), 453-466.
- National Guideline Centre (UK). Preoperative Tests (Update): Routine Preoperative Tests for Elective Surgery. London: National Institute for Health and Care Excellence (NICE); 2016 Apr. (NICE Guideline, No. 45.) Acknowledgements.
- Practice Advisory for Preanesthesia Evaluation: An Updated Report by the American Society of Anesthesiologists Task Force on Preanesthesia Evaluation. Anesthesiology 2012; 116:522–538 doi: https://doi.org/10.1097/ALN.0b013e31823c1067

Version 1.4 February 2024

© 2025 Mott NM et al. *JAMA Network Open*.

Appendix 2:

Primary Care Physician (PCP) Letter

© 2025 Mott NM et al. *JAMA Network Open*.

RITE-Size
RIGHT-SIZING TESTING BEFORE ELECTIVE SURGERY

Insert your institutional logo here

(delete this box after)

Greetings (Insert site name here),

I am writing on behalf of (insert your institution-department here) to make your practice aware of ongoing efforts to reduce unnecessary preoperative testing before low-risk surgeries. We felt it would be beneficial to share some information with you given our dual roles in preparing our patients for a safe surgery.

Our site is engaging with multiple state-wide collaborative quality improvement teams (ASPIRE/MPOG, MPrOVE, MSQC, MVC) to decrease rates of unnecessary preoperative testing before low-risk surgery (e.g., breast lumpectomy, inguinal hernia repair, and laparoscopic cholecystectomy). There is overwhelming evidence that routinely performing preoperative tests (e.g., blood tests, EKGs, urine tests, chest x-rays, and stress tests) on patients undergoing low-risk surgery does not improve outcomes, may be misinformative and introduce delays, and wastes healthcare resources.

We encourage you to review the resources on the following page and implement them within your preoperative clearance practice for patients referred for one of the low-risk surgeries mentioned above. All of these resources are evidence-based and derived from multiple national societies' recommendations. Of course, these decision tools do not override your own medical decision-making; they are guidelines to aid in making coordinated preoperative testing decisions within our care teams.

If you have any questions, please reach out to me or the contacts provided on the next page.

Thank you for your consideration and assistance as we work to reduce low-value care in our hospital and the state of Michigan. Please share widely within your practice as appropriate.

Best,

Your Name
Role, Institution

© 2025 Mott NM et al. *JAMA Network Open*.

RITE-Size: Right-Sizing Testing Before Elective Surgery

Frequently Asked Questions:

Who are ASPIRE/MPOG, MPrOVE, MSQC, and MVC?

- **MPOG: Multicenter Perioperative Outcomes Group:** MPOG consists of over 70 participating hospitals who share their mission to promote safe and evidence-based perioperative care for all patients through collaboration, research, education, and quality improvement.
- **MPrOVE: Michigan Program on Value Enhancement:** MPrOVE works to identify, design, and rigorously and rapidly evaluate specific projects focused on improving quality and demonstrating the value of clinical services at Michigan Medicine and beyond.
- **MSQC: Michigan Surgical Quality Collaborative :** A collaborative of Michigan hospitals dedicated to overall surgical quality improvement, including better patient care and lower costs. They host a robust regional registry to analyze the issues, identify the best practices, and disseminate them widely.
- **MVC: Michigan Value Collaborative:** A partnership between Michigan hospitals, physician organizations, and BCBSM/BCN. MVC uses claims data to understand variation in healthcare use and identify best practices, and supports statewide collaboration on interventions that improve the value of care before, during, and after hospitalization.

Why was this letter sent to your practice? / Why is it important for PCPs to be engaged?

- Clearing a patient for surgery is a team effort with the shared goal of patient safety in mind. Over-ordering tests can often happen when providers aren't on the same page with one another about what is necessary. Our team has adopted a testing policy and decision aid based on national guidelines that will help expedite the preoperative process while preventing the potential drawbacks of unnecessary testing. We want you to be aware of these decisions and recommendations as part of the care team.
- We know some preoperative testing orders may come from your office when patients are referred for preoperative clearance or other preoperative appointments. The surgical and anesthesia teams will be following the enclosed guidance; there is no need to order additional testing unless you deem it clinically necessary.

What are the target procedures and tests?

- This initiative focuses on reducing tests before breast lumpectomy, inguinal hernia repair, and laparoscopic cholecystectomy for internal data reasons, but the same approach is also appropriate for other common low-risk procedures.
- The tests of interest include blood tests (e.g., CBC, BMP, CMP, PT, PTT), EKGs, urine tests, chest X-rays, and stress tests.

What if providers still want to order preoperative tests?

- Individual decisions about testing will remain at the discretion of the treating clinicians. The intent of sharing these resources is to provide *guidelines* and decision support in use by our surgical and anesthesia care teams regarding specific preoperative tests for low-risk surgical cases (as defined by both the surgical risk and patient risk).

Questions?

If you have questions, please contact the faculty leads or project managers for this initiative.
Faculty Leads: Hari Nathan (MVC), drnathan@umich.edu; Lesly Dossett (MPrOVE), ldossett@med.umich.edu; Anthony Edelman (MPOG), aedelman@med.umich.edu
RITE-Size Project Manager: Dana Greene, greenejr@med.umich.edu
Learn more at our website: RiteSizeTesting.org

© 2025 Mott NM et al. *JAMA Network Open*.

Insert your hospital logo here

This resource was developed in partnership with MPOG, MPrOVE, MSQC, and MVC.

Suggested Preoperative Testing Decision Aid for Low-Risk Surgeries

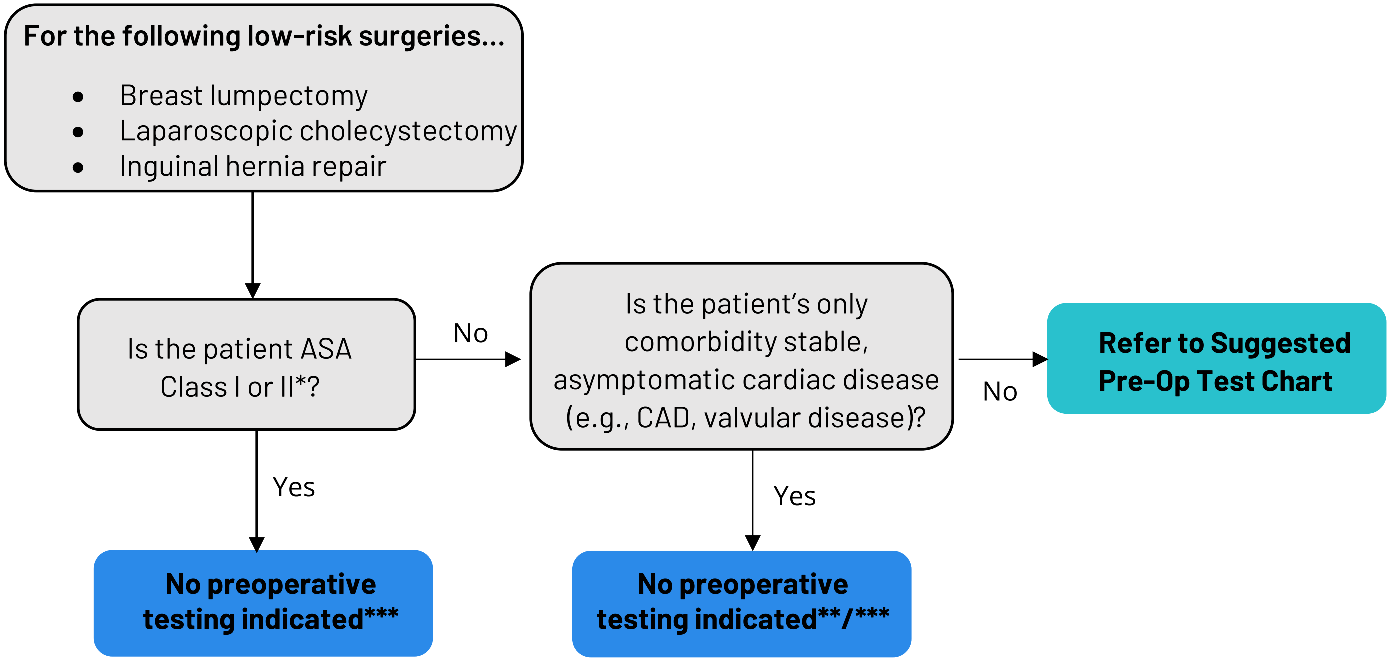

***American Society of Anesthesiologist (ASA) Physical Status Classification System:**

ASA Class I: Normal healthy patient. Non-smoking, no or minimal alcohol use, no acute or chronic disease, normal BMI.

ASA Class II: Mild systemic disease without substantive functional limitations. Current smoker, obesity (30<BMI<40), well-controlled DM/HTN, mild lung disease.

ASA Class III: Severe Systemic disease with substantive functional limitations, poorly controlled DM/HTN, COPD, morbid obesity (BMI ≥ 40), active hepatitis, alcohol dependence or abuse, pacemaker, moderate reduced EF, ESRD on dialysis, prior MI, CVA, TIA, or CAD/stents > 3 months ago

**May consider EKG if none available within the past ~6 months

***Except recent (3-6 months) potassium if on diuretic

All recommendations in this document pertain to non-pregnant, adult patients undergoing low-risk procedures. They do not replace clinical judgment and are intended as guidance only.

Version 1.4 February 2024

© 2025 Mott NM et al. *JAMA Network Open*.

Insert your hospital logo here

This resource was developed in partnership with MPOG, MPrOVE, MSQC, and MVC.

Suggested Preoperative Tests for Patients Undergoing Low-Risk Surgery Who Are ASA III or Above

This chart does not replace clinical judgment and is intended as guidance only

	CBC	T&S	BMP	LFTs	INR/PT/PTT	EKG
History of anemia, thrombocytopenia	X					
Cardiovascular disease	X					X
Anticoagulant use or history of bleeding disorder	X	X			X	
DM/major endocrine disease, prior electrolyte abnormalities, use of diuretics, antiarrhythmics, ACE/ARB			X			
Kidney disease	X		X			
Liver disease or risk of malnutrition			X	X	X	
Peripheral/cerebral vascular disease, cardiac risk factors, new cardiac symptoms			X			X

CBC: Complete blood count

T&S: Type and screen

BMP: Basic metabolic panel

LFTs: Liver function tests

INR: International normalized ratio

PT: Prothrombin time

PTT: Partial thromboplastic time

EKG: Electrocardiogram

ACE: Angiotensin-converting enzyme inhibitors

ARB: Angiotensin receptor blocker

References:

- Mocon A, McRitchie D, Tharani A. *Drop the Pre-Op: A toolkit for reducing unnecessary visits and investigations in pre-operative clinics.* Ontario, CA. 2019.
- Chow, W. B., Rosenthal, R. A., Merkow, R. P., Ko, C. Y., & Esnaola, N. F. (2012). Optimal preoperative assessment of the geriatric surgical patient: a best practices guideline from the American College of Surgeons National Surgical Quality Improvement Program and the American Geriatrics Society. *Journal of the American College of Surgeons*, *215*(4), 453-466.
- National Guideline Centre (UK). Preoperative Tests (Update): Routine Preoperative Tests for Elective Surgery. London: National Institute for Health and Care Excellence (NICE); 2016 Apr. (NICE Guideline, No. 45.) Acknowledgements.
- Practice Advisory for Preanesthesia Evaluation: An Updated Report by the American Society of Anesthesiologists Task Force on Preanesthesia Evaluation. Anesthesiology 2012; 116:522–538 doi: https://doi.org/10.1097/ALN.0b013e31823c1067

Version 1.4 February 2024

© 2025 Mott NM et al. *JAMA Network Open*.

Appendix 3:

Interview Guide

© 2025 Mott NM et al. *JAMA Network Open*.

RITE-Size (Right Sizing Pre-Operative Testing Before Low-Risk Surgery)

Interview Guide_Final

Introduction:

1. Hello, my name is (**insert name**) and this is my partner (**insert name**). We will be conducting a 30-minute interview with you today. You were recommended by your Quality Lead **(insert name**) to participate in today's interview. Can you please verbally state your name, position and confirm your willingness to participate in this interview.
   a. *Acknowledge participants consent. If the participant is refusing to provide verbal acknowledgement, please end the interview and thank them for their time.*
   b. *If participant has provided verbal acknowledgment, please proceed with the interview.*
2. Thank you for agreeing to participate in this interview. At this time, I would like to begin recording for the purposes of our study records and data collection. At this point, are you fine with me recording this session.
   a. *Confirm consent to record and proceed with recording.*
3. At the end of this interview, you will be asked to complete a brief survey.

Stakeholder Engagement:

1. Can you please state your **name, title, and number of years** you've worked at this hospital in your current position?
2. Can you please describe your role at the hospital?
   a. *What surgeries do they perform?*
   b. *What's the typical case volume of procedures? (rough estimate)*
   c. *How do they interact with other team members?*
3. What is the current size of your team?
   a. Are there members of your team who are not regularly onsite (part-time employees, on-call surgeons or anesthesiologist).
4. As a member of (**insert team**) does you day to day work include reviewing patient charts, scheduling surgeries, or ordering pre-operative testing?
   a. If yes: Can you describe your role?
   b. If no: Whose role does that primarily fall to?
5. Are you aware of the strategies/or initiatives your hospital has deployed to reduce low-value pre-operative testing?
   a. *If yes, please ask them to list the strategies*
   b. *If not, state the following: Since (date) your site has engaged in several strategies (list).*

© 2025 Mott NM et al. *JAMA Network Open*.

Consensus Process:

1. As you understand it, can you please describe the motivation to reduce low value pre-operative testing at your hospital?
2. You listed the following strategies (**insert strategies**). How did your team decide what strategies to use?
    a. *What worked well during this process?*
    b. *What challenges existed during this process?*
    c. *How were these challenges resolved?*
    d. *If you could engage in the process again, what would you do differently, if anything?*
3. Are there any strategies you're still working on or trying to change?
    a. *How were RITE-Size pre-operative testing resources/components modified to work better at your hospital?*

Strategy Implementation:

If the site used more than one strategy, please ask them to specify for each question.

1. Once strategies were selected for implementation how was the staff informed of this decision?
2. Did staff receive training ?
   If so:
    a. How was the training received?
    b. What worked well during the training?
    c. What, if any, challenges existed during the staff training?
    d. Were these challenges anticipated?
    e. How were these challenges resolved?
3. Can you please describe how the strategy/strategies was/were implemented?
    a. What worked well during strategy implementation?
    b. What, if any, push back existed during the strategy implementation?
    c. Were these challenges anticipated?
    d. How were these challenges resolved?
4. Does your hospital plan to continue using this strategy in the future?
    a. How are you planning to sustain the practice?

Implementation Coaching:

This section should be reserved for our quality lead. The quality lead should be the last person we interview from our sample.

1. Can you please describe how participation and RITE-Size implementation coaching session helped you implement strategies to reduce low-value pre-operative testing, if at all?
2. What component of the RITE-Size intervention was most beneficial to helping reduce low-value pre-operative testing?

© 2025 Mott NM et al. *JAMA Network Open*.

3. What, if anything, could be added to the RITE-Size intervention to support the reduction of testing rates?
4. Were there any components of the RITE-Size intervention that you felt did not assist you in reducing testing rates?
5. Is there anything the RITE-Size team could have done differently to help you implement strategies?

Conclusion:

1. This concludes our interview. Once, again I'd like to thank you for your participation in the RITE-Size intervention. The RITE-Size staff will be available to help you answer any questions or concerns regarding strategy implementation or unanticipated barriers over the next four weeks.
2. We are posting a link to a survey in the chat. This should take no longer than 5 minutes. Can you please complete the survey? Thank you

© 2025 Mott NM et al. *JAMA Network Open*.

Appendix 4:

Survey

© 2025 Mott NM et al. *JAMA Network Open*.

Part 1: All materials

Please answer these questions considering the RITE-Size intervention holistically.

The RITE-Size intervention holistically includes the site visit, implementation coaching sessions, collaborative review of data, consensus process, and development of strategies, which may include decision aids, modification of anesthesia protocols, and auditing/feedback systems. The RITE-Size intervention also includes resources to help with the deployment and uptake of these strategies at your site.

Acceptability of Intervention Measure (AIM)

© 2025 Mott NM et al. JAMA Network Open.

	Completely disagree	Disagree	Neither agree nor disagree	Agree	Completely agree
1. The intervention meets my approval.	○	○	○	○	○
2. The intervention is appealing to me.	○	○	○	○	○
3. I like the intervention.	○	○	○	○	○
4. I welcome the intervention.	○	○	○	○	○

Intervention Appropriateness Measure (IAM)

	Completely disagree	Disagree	Neither agree nor disagree	Agree	Completely agree
1. The intervention seems fitting.	○	○	○	○	○
2. The intervention seems suitable.	○	○	○	○	○
3. The intervention seems applicable.	○	○	○	○	○
4. The intervention seems like a good match.	○	○	○	○	○

© 2025 Mott NM et al. *JAMA Network Open*.

Part 2: RITE-Size intervention process

Please rate your agreement with the following statement considering the RITE-Size intervention components individually.

"This intervention component was helpful in reducing pre-operative testing."

	Completely disagree	Disagree	Neither agree or disagree	Agree	Completely agree	Not applicable
Site visit	○	○	○	○	○	○
Implementation coaching sessions	○	○	○	○	○	○
Collaborative review of Michigan Surgical Quality Collaborative (MSQC) or Michigan Value Collaborative (MVC) Data	○	○	○	○	○	○
Consensus process (i.e., development of strategies to reduce pre-operative testing)	○	○	○	○	○	○

© 2025 Mott NM et al. JAMA Network Open.

	Completely disagree	Disagree	Neither agree or disagree	Agree	Completely agree	Not applicable
Strategy deployment (i.e., resources to support distribution and uptake of strategies)	○	○	○	○	○	○

Part 3: RITE-Size strategy components

Which strategies did you adopt in an effort to reduce pre-operative testing? Please choose ALL that apply.

- ☐ Decision aid
- ☐ Modification of anesthesia or pre-operative protocol
- ☐ Auditing/feedback system
- ☐ [ ] Other, please specify:

Of the strategies you utilized, which were helpful in reducing pre-operative testing? (Drag and drop the individual items to the corresponding group/bin. More than one item can be placed in a group/bin.)

© 2025 Mott NM et al. JAMA Network Open.

Items
Decision aid
Modification of anesthesia pre-operative protocol
Auditing/feedback system
Other, please specify:

Most helpful

Helpful

Neither helpful or unhelpful

Unhelpful

Least helpful

© 2025 Mott NM et al. *JAMA Network Open*.

Part 4: Decision aid

You indicated that using a decision aid was one of the strategies you employed.

Please answer these questions considering the **decision aid** specifically.

Acceptability of Intervention Measure (AIM)

	Completely disagree	Disagree	Neither agree nor disagree	Agree	Completely agree
1. The decision aid meets my approval.	○	○	○	○	○
2. The decision aid is appealing to me.	○	○	○	○	○
3. I like the decision aid.	○	○	○	○	○

© 2025 Mott NM et al. *JAMA Network Open*.

	Completely disagree	Disagree	Neither agree nor disagree	Agree	Completely agree
4. I welcome the decision aid.	○	○	○	○	○

Intervention Appropriateness Measure (IAM)

	Completely disagree	Disagree	Neither agree nor disagree	Agree	Completely agree
1. The decision aid seems fitting.	○	○	○	○	○
2. The decision aid seems suitable.	○	○	○	○	○
3. The decision aid seems applicable.	○	○	○	○	○
4. The decision aid seems like a good match.	○	○	○	○	○

Part 5: Protocol modification

You indicated that modifying the anesthesia or pre-operative protocol was one of the strategies you employed.

© 2025 Mott NM et al. *JAMA Network Open*.

Please answer these questions considering the **protocol modification** specifically.

Acceptability of Intervention Measure (AIM)

	Completely disagree	Disagree	Neither agree nor disagree	Agree	Completely agree
1. The protocol modification meets my approval.	◯	◯	◯	◯	◯
2. The protocol modification is appealing to me.	◯	◯	◯	◯	◯
3. I like the protocol modification.	◯	◯	◯	◯	◯
4. I welcome the protocol modification.	◯	◯	◯	◯	◯

Intervention Appropriateness Measure (IAM)

	Completely disagree	Disagree	Neither agree nor disagree	Agree	Completely agree
1. The protocol modification seems fitting.	◯	◯	◯	◯	◯

© 2025 Mott NM et al. *JAMA Network Open*.

	Completely disagree	Disagree	Neither agree nor disagree	Agree	Completely agree
2. The protocol modification seems suitable.	○	○	○	○	○
3. The protocol modification seems applicable.	○	○	○	○	○
4. The protocol modification seems like a good match.	○	○	○	○	○

Part 6: Auditing/feedback

You indicated that using an auditing or feedback system was one of the strategies you employed.

Please answer these questions considering the **auditing or feedback system** specifically.

Acceptability of Intervention Measure (AIM)

© 2025 Mott NM et al. *JAMA Network Open*.

	Completely disagree	Disagree	Neither agree nor disagree	Agree	Completely agree
1. The auditing or feedback system meets my approval.	○	○	○	○	○
2. The auditing or feedback system is appealing to me.	○	○	○	○	○
3. I like the auditing or feedback system.	○	○	○	○	○
4. I welcome the auditing or feedback system.	○	○	○	○	○

Intervention Appropriateness Measure (IAM)

	Completely disagree	Disagree	Neither agree nor disagree	Agree	Completely agree
1. The auditing or feedback system seems fitting.	○	○	○	○	○
2. The auditing or feedback system seems suitable.	○	○	○	○	○
3. The auditing or feedback system seems applicable.	○	○	○	○	○

© 2025 Mott NM et al. *JAMA Network Open*.

	Completely disagree	Disagree	Neither agree nor disagree	Agree	Completely agree
4. The auditing or feedback system seems like a good match.	○	○	○	○	○

Part 7: Demographics

Please answer these brief demographic questions to complete the survey.

What hospital do you work at?

- ○ Trinity Health Grand Haven
- ○ ProMedica Charles and Victoria Hickman Hospital
- ○ University of Michigan Health - Sparrow Lansing

What is your profession?

- ○ Medicine physician (e.g., family medicine, internal medicine, or other primary care provider responsible for ordering tests)
- ○ Anesthesiologist

© 2025 Mott NM et al. *JAMA Network Open*.

- General surgeon
- Nurse
- Administrator
- Other profession, please specify:

If you are able to receive a check ($150) for participating in this interview, please write your name and mailing address in the space provided so that we may send you the study compensation.

© 2025 Mott NM et al. JAMA Network Open.
