# Supplementary material for: Right-Sizing Testing Before Elective Surgery for Patients With Low Risk
Source: JAMA Netw Open. 2025 Oct 6;8(10):e2535750. doi: 10.1001/jamanetworkopen.2025.35750 (PMC12501813; doi:10.1001/jamanetworkopen.2025.35750)
Supplement: Supplement 2. — Data Sharing Statement [file jamanetwopen-e2535750-s002.pdf]

Data Sharing Statement

Mott NM. Right-Sizing Testing Before Elective Surgery for Patients With Low Risk. *JAMA Netw Open*. Published October 06, 2025. doi:10.1001/jamanetworkopen.2025.35750

Data
Data available: No

Additional Information
Explanation for why data not available: The de-identified data supporting the findings of this study are available from the corresponding author upon reasonable request but will not be made publicly available.
